# Dendritic cells in inborn errors of immunity

**DOI:** 10.3389/fimmu.2023.1080129

**Published:** 2023-01-23

**Authors:** Sudhir Gupta, Anshu Agrawal

**Affiliations:** Division of Basic and Clinical Immunology, University of California, Irvine, CA, United States

**Keywords:** dendritic cells, inborn errors of immunity, primary immunodeficiencies, plasmacytoid DCs, myeloid DCs, classical DCs, monocyte derived DCs

## Abstract

Dendritic cells (DCs) are crucial cells for initiating and maintaining immune response. They play critical role in homeostasis, inflammation, and autoimmunity. A number of molecules regulate their functions including synapse formation, migration, immunity, and induction of tolerance. A number of IEI are characterized by mutations in genes encoding several of these molecules resulting in immunodeficiency, inflammation, and autoimmunity in IEI. Currently, there are 465 Inborn errors of immunity (IEI) that have been grouped in 10 different categories. However, comprehensive studies of DCs have been reported in only few IEI. Here we have reviewed biology of DCs in IEI classified according to recently published IUIS classification. We have reviewed DCs in selected IEI in each group category and discussed in depth changes in DCs where significant data are available regarding role of DCs in clinical and immunological manifestations. These include severe immunodeficiency diseases, antibody deficiencies, combined immunodeficiency with associated and syndromic features, especially disorders of synapse formation, and disorders of immune regulation.

## 1 Introduction

Dendritic cells (DCs) are the main antigen-presenting sentinels of the immune system whose role in T lymphocyte activation facilitates cross-communication between innate and adaptive immunity (1). While present in virtually all tissues, dendritic cells only comprise 0.1-1% of the mononuclear cell (MNC) population in peripheral blood (2). Human DCs develop from bone marrow and DC progenitors but can also be derived from monocytes, characteristically expressing high levels of cell surface marker CD11c and MHC class II (3, 4). DCs sense and respond to pathogens *via* pattern recognition receptors (PRRs). The most prominent amongst the PRRs are the Toll like receptors (TLRs) that are present both as intracellular and cell-surface proteins (5). In addition to TLRs, other PRRs include Nod like receptors (NLRs), C-type lectin receptors (CLRs) and RIG-1 like receptors (RLRs) (6). PRRs bind highly conserved microbial structures, known as pathogen-associated molecular patterns (PAMPs). Together all the PRRs in humans can detect anything of foreign origin including single and double stranded nucleic acids, lipoproteins, peptidoglycans, other microbial components as well as sugars (5, 6). The PRRs also recognize endogenous ligands that are released in the body during injury, stress etc. known collectively as danger associated molecular patterns (DAMPs). DCs in quiescent state before activation are known as immature DCs. Immature DCs are geared towards antigen capture and thus have high endocytic capacity *via* the PRRs as well as *via* macropinocytosis. However, the immature DCs express low levels of costimulatory molecules and chemokine receptors and are poor at priming T cells. Upon pathogen recognition, DCs become activated or mature. The primary function of mature DCs is to present antigen to T cells, thus the expression of costimulatory molecules, MHCs and inflammatory cytokines is upregulated. The mature DCs also upregulate chemokine receptors such as CCR7 that allows them to migrate to nearby lymph nodes to present the MHC-peptide complex to antigen-specific CD4^+^ and CD8^+^ T cells *via* T-cell receptor (TCR) binding (7). Successful binding induces tyrosine phosphorylation to initiate gene transcription of various pro- and anti-inflammatory cytokines. Such mediators further activate and proliferate naive T-lymphocytes to promote a stronger host adaptive immune response.

The activation of DCs *via* various PRRs induces distinct patterns of cytokine/inflammatory mediators’ secretion which in turn dictates the nature of T cell responses (8). For example, activation of DCs *via* TLR4 induces the production of IL-12p70 from DCs and IL-12 acts on CD4 T cells to bias the T cell response towards IFN-γ secreting Th1 cells. Similarly, activation of DCs *via* Dectin-1 induces a Th17 bias while when both Dectin-1 and TLR2 are activated together as in the case of yeast Zymosan, the Th response is a mixture of Treg/Th17 (9). Viruses are recognized primarily by intracellular receptors such as TLR7, TLR9, RIG-I, and MDA-5 and lead to the production of type-I IFNs, which in turn induce CTLs and B cell antibody production (10–12). In contrast to TLRs, inflammasomes are activated by primarily by colloidal or particulate matter (exogenous or endogenous) such as Alum or Uric acid and result in the production of highly inflammatory cytokines, IL-1β, IL-18, IL-33 etc. that have been shown to be involved in several inflammatory diseases (13, 14). DCs are therefore essential in defining the nature of T cell responses.

**Figure 1:**
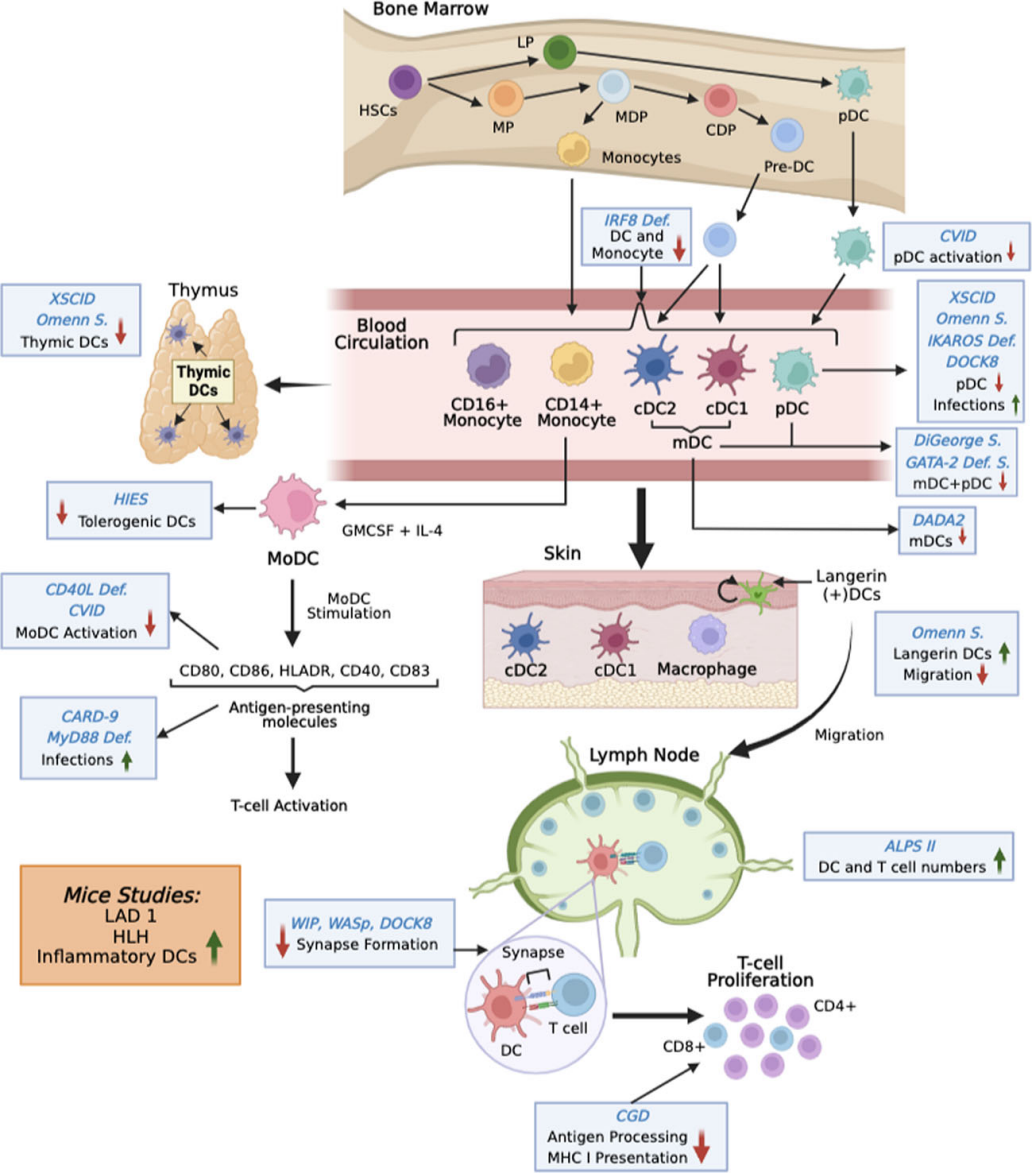
Overview of changes in DC functions in IEI. DCs develop in the bone marrow from hematopoietic stem cells (HSC). After differentiation into preDCs (for mDCs) and pDCs they leave the bone marrow and enter into the circulation. From there they populate different tissues. Monocytes can also differentiate into DCs. The figure shows changes in DCs at various stages in different IEIs (blue Italics). S is syndrome. Mutations studied in mice are shown in orange box.

Several peripheral blood DC subsets have been well-characterized in humans that help direct the immune response toward different avenues. The three major subsets of DCs present in the blood are the conventional (classical) dendritic cells(cDCs), also known as myeloid DCs (mDCs). cDCs can be divided into the cDC1 and cDC2 subpopulations (15, 16) (**Figure 1**). The other subset is the plasmacytoid DC (pDC) subset. As is the case of other immune cells DCs also develop from CD34^+^ progenitor cells. The CD34^+^ progenitors give rise to lymphoid and myeloid precursors. The myeloid precursors then differentiate into monocytes and common DC precursors. All three DC subsets are derived from a common DC progenitor in the bone marrow. The common DC progenitor differentiates directly into pDCs and pre-DCs both of which are released into the circulation (17). The two cDC subsets found in circulation and peripheral lymphoid organs stem from pre-DCs.

The cDC1 and cDC2 subpopulations are both capable of activating naive T lymphocytes, cDC1s express almost exclusively cell adhesion molecule 1 (CADM1) and high levels of C-type lectin-like receptor (Clec) 9A, CD141, and XCR1 on its surface. Expression of key transcription factors such as DNA-binding protein inhibitor 2 (ID2), interferon regulatory factor 8 (IRF8), and BATF3 are hallmarks of cDC1. cDC1 are resistant to viral infections. They are also known for their superior ability to cross-present and prime CD8^+^ cytotoxic T lymphocytes against exogenous antigens, particularly in mice. Cross-presentation provides the ability to DCs to prime CD8+T cells responses against viruses and tumors even in the absence of self-infection (17, 18). In contrast, cDC2s are characterized by high expression of CD1c, IRF4, Notch2, and KLF4, ZEB2, ETS2. The cDC2 subset is distinguished by greater expression of MHC class II genes and thus are extremely efficient in CD4^+^ T cell priming (17). Though cDC1’s are considered the major cDC subset responsible for cross-presentation as determined by mice studies nevertheless in humans the cDC2’s are equally efficient at cross-presenting antigens to CD8 T cells.

The differentiation of pDC’s is completed in the bone marrow. In the circulation they are distinguished by the expression of CD123, CD303 and CD304 and absence of CD11c. In an immature state, pDCs are incapable of activating a T cell response, but once mature are best characterized for producing high levels of type I and III interferon upon viral infections. They are involved in NK cell activation *via* secretion of cytokines IL-12 and IL-18. Additionally, pDCs express high levels of intracellular TLR7 and TLR9. TLR7 can recognize single-stranded RNA viruses to induce NK-kB expression, leading to pro-inflammatory TNF-alpha and IL-6 production (5, 16, 19).

Another major population of DCs that is most well studied is the monocyte derived DC subset (MoDCs) (20). MoDCs like cDC2 require IRF4 for differentiation (21). They also express high levels of CD11c and MHC-II and considered inflammatory DCs. The MoDCs are usually derived *in vitro* but studies indicate that MoDCs can also be derived *in vivo* under inflammatory conditions (17). The MoDCs have been observed in the lungs during COVID-19 infection, in the skin in atopic dermatitis patients as well as in the synovial fluid of rheumatoid arthritis patients (22–24). Because of the ease of deriving MoDCs *in vitro* in large quantities, they are the primary DC subset used for immunotherapy in tumors.

The advent of single cell RNA sequencing has led to the discovery of several novel cDC and pDC subsets, many of which appear to be transient in nature (25). In a recent discovery, Axl^+^ DCs which are CD123^+^ myeloid DCs have shown to possess a combination of pDC-like and cDC2-like properties (25). While they can span a wide range of properties, their basic function is most similar to cDC2s. They can respond to bacterial infections *via* TLR4 and TLR5 expression like cDC2s but unable to produce IFN-I upon TLR7/9 activation like pDCs. For both subsets more research is needed to elucidate their relevance to immune function.

## 2 Dendritic cells in inborn errors of immunity

Inborn errors of immunity (IEI) are a group of disorders clinically present with increased susceptibility to infections, autoimmunity, allergic diseases, autoinflammation, and/or malignancies. There are 485 IEI and more than 400 genes causing these disorders that have been described (26). Since DCs are critical antigen presenting cells that prime T cells and play an important role in immune homeostasis (27); alterations in number and functions may contribute to mechanisms responsible for increased susceptibility to infections, autoimmunity and autoinflammation in IEI. IEI have been grouped into 10 different categories. DCs have not been studied in detail in majority of IEI. In this review, we will present analysis of DCs in common disorders in each category of IEI except complement deficiencies in which no study of DCs has been reported.

## 3 Dendritic cells in Immunodeficiencies affecting cellular and humoral immunity

Most severe of primary immunodeficiencies are severe combined immunodeficiency (SCID) results from mutations in genes regulating lymphopoiesis. SCID consist mainly characterized by defects in both T- and B-lymphocyte function, with variable defects in natural killer (NK) cell cytolytic activity.

### 3.1 DCs in T-B+ severe combined immunodeficiency

#### 3.1.1 SCID due to γc mutation

X-linked SCID is the most common SCID caused by mutations in IL-2 receptor gamma chain gene (*IL2-RG*) (28). IL-2RG is expressed on all hematopoietic cells, and shared by the receptors of IL-2, IL-4, IL-7, IL-9, IL-15, and IL-21 (29). Patient present with T-B-NK+ phenotype and functionally defective B cells. Thymic DCs are markedly reduced in X-SCID (30, 31). Hale et al. (31) reported >50% reduction in CD83+ DCs in the thymus in X-SCID. Patients with milder phenotype of X-SCID (atypical X-SCID) with rare hemizygous *IL-2RG* mutation have been described (32, 33) Tuovinen and colleagues (34) reported markedly decreased pDCs in a patient with IL-2RG hemizygous mutation. This could be due to defective signaling *via* IL-4 and IL-15; these two cytokines are survival factors for DCs (35). Shultz et al. (36), in NOD-*scid IL-2Rγ* ^null^ mice, observed reduced *in vitro* generation of TNF+ and CD86+ DCs. Following engraftment with mobilized human hematopoietic stem cells, pDCs along with T cells, B cells, and NK cells are reconstituted.

### 3.2 DCs in T-B-SCID

#### 3.2.1 *RAG* mutation immunodeficiency

Recombination-activating genes (*RAG*)*1* and *RAG2* encode endonuclease proteins, initiate the process of V(D)J recombination that leads to lymphocyte receptor formation, and therefore play an essential role in adaptive immunity. RAG deficiency may manifest with a broad range of phenotypes that reflect the degree of adaptive immunity compromised which in turn depends, at least in part, on the residual recombination activity of the mutant RAG protein(s). Nonsense mutations in either *RAG1* or *RAG2* result in the failure of the initiation of antigen receptor recombination, causing a very similar phenotype with complete lack of mature T and B cells resulting in severe combined immunodeficient (T^−^B-NK+SCID) phenotype, both in humans and mice (37–39). Infants classically present with infectious symptoms caused by common viral pathogens (40–42), susceptibility to opportunistic pathogens such as *Pneumocystis jirovecii*, and failure to thrive.

##### 3.2.1.1 Omenn syndrome

Omenn syndrome is a rare autosomal recessive severe combined immunodeficiency due to RAG deficiency characterized by prominent immune dysregulation, with erythroderma, lymphadenopathy, hepatosplenomegaly, eosinophilia, and elevated IgE but low IgG, IgA, and IgM. T-lymphocytic infiltrates are present in target organs, contributing to tissue damage. It is characterized by T+B-NK- phenotype; T cells are oligoclonal with restricted T cell repertoire, extremely reduced B cells, hypogammaglobulinemia, elevated IgE, and immune dysregulation (43, 44). A majority of thymic DCs subpopulations (S-100+ CD11c+ and CD303/BDCA2+ are severely reduced or completely lacking (30); however, DCs are present in the skin (45). Langerin+ cells are increased and abnormally distributed in the derma as compared to healthy controls (46). Maina et al. (46) examined DC homeostasis and functions in RAG2R229Q mouse model of Omenn syndrome and also observed that majority of Langerin+ cells were increased and were present in dermis of mutant mice with severe erythroderma. In the lymph nodes, CD11c+ DCs were similar between mutant mice and RAG+/+ mice in the steady state; however, under inflammatory conditions number of CD11c cells were reduced. This would suggest defective migration of Langerin+ cells from the skin to lymph nodes. This in contrasts with the bone marrow DCs that were similar in functions between mutant and RAG+/+ mice with regard to LPS-induced production of IL-12-p70, proliferation of naïve allogeneic T cells, and expression of CCL3, CCL5, and CCL-19. In the thymus of RAG2R229Q mice number and frequency of pDCs and CD8+cDCs are significantly reduced.

The first autosomal recessive defect causing SCID identified was due to a defect in the gene encoding adenosine deaminase (ADA) gene, resulting in intracellular cellular accumulation of adenosine and its precursors, leading to apoptosis of both T and B cells (47).

#### 3.2.2 Adenosine Deaminase A2 deficiency

DADA2 is caused by biallelic non-sense mutation in ADA2 resulting in the clinical phenotype of recurrent fevers, vasculitis, immunodeficiency (pancytopenia) and cytopenia (48–50). Yap et al. (51) performed extensive phenotypic studies in 110 patients with DADA2. CD123+CD11c− pDCs, and CD123−CD11c+ conventional DCs) were comparable in frequencies in DADA2 patients and healthy controls. However, when data were analyzed for two subsets of conventional DCs, DADA2 patients had significantly reduced proportions of CD1chi and CD141+ conventional subsets as compared to healthy controls. Therefore, ADA2 deficiency does not appear to effect on overall numbers and proportions of dendritic cells; however, it does alter the distribution of mDC subsets

#### 3.2.3 IKAROS deficiency

IKAROS Family Zinc Finger 1 (IKZF1 or IKAROS) was first identified in mice to play an important role in the differentiation and development of T and B lymphocytes and natural killer (NK) cells (52). IKAROS, in addition to be essential for myeloid, megakaryocyte, and erythroid differentiation is also required for pDCs (53). They observed that mice expressing low levels of IKAROS) lack peripheral pDCs, but not other DC subsets. Furthermore, loss of pDCs is associated with an inability to produce type I IFN after challenge in response to TLR-7 and TLR-9. In humans, first case of *IKZF1* mutation was reported in an infant with complete loss of B cells, very low NK cells, and remaining lymphocytes were reactive T cells (99%); however, with impaired T cell proliferation upon mitogens and TCR stimulants (54). Since then, 90 cases have been described with three distinct types of variants. IKAROS Family Zinc Finger 1 (IKZF1 or IKAROS) heterozygous mutation present with hypogammaglobulinemia and progressive B cell deficiency. pDCs are reduced, whereas cDCs are either normal or expanded (55). The clinical and immunological phenotypes of patients carrying IKZF1 mutations appear to depend on the domain primarily affected by the mutation (i.e., DNA binding or dimerization) and the mechanism of action involved (i.e., haploinsufficiency or dominant negative) impacting IKAROS functions. Patients with DN mutations display most severe form of disease, whereas patients with DD mutations present with mild form of disease. Patients with HI mutations have mostly bacterial infections manifesting as Pneumonia, otitis, sinusitis, bronchitis, and meningitis. Patients with DN mutations suffer from severe fungal, bacterial, and viral infections. The majority of patients with IKAROS-associated diseases have B cell development and B cell function defects, and low levels of immunoglobulins.

#### 3.2.4 CD40 ligand deficiency

X-linked hyper IgM syndrome (HIGM1) is caused by mutation of CD40L (CD154) and characterized by bacterial and opportunistic infections including *Candida albicans and Paracoccidioides brasiliensis.* Cabral-Marques and colleagues (56). studied DCs phenotypically and functionally in 5 patients with HIGM1. iDCs obtained by differentiation of monocytes with GM-CSF and IL-4 expressed reduced levels of co-stimulatory CD80, CD86, and HLA-DRII as compared to controls; however, expressed normal levels of TLR2 and TLR4. They also reported that stimulation of iDCs with *C. albicans and P. brasiliensis* resulted in reduced expression of CD80 and HLA-DR as compared to controls. These observations are similar to those reported by Fontana and colleagues (57) in 2 patients with hypomorphic mutation of CD40 (HIGM 3). Incubation of iDCs with sCD154 resulted in upregulation of CD80, CD86, and HLA-DR to the levels of controls. The percentage of TH17 cells and IL-17 production were normal when patient’s DCs were co-culture with autologous T cells or MNC cells from patients were stimulated with PMA and ionomycin, or T cells were stimulated with *C. albicans* pulsed DCs. Therefore, excluding a possibility of a role of IL-17 in increased susceptibility to fungal infections in HIGM 1. DCs pulsed with *C. albicans and P. brasiliensis* produced significantly reduced IL-12 and increased IL-10 as compared to controls. Several investigators have also reported decreased IL-12 and increased IL-10 upon stimulation by DCs pulsed with a variety of microbes (58, 59). Furthermore, DCs pulsed with *C. albicans and P. brasiliensis* when co-cultured with T cells resulted in reduced IFNγ production (TH1) and increased IL-4 and IL-5 production (TH2). This pattern was reversed by exogenous sCD154. Fontana et al. (57) also observed defect in IFNγ production by allogeneic T cells induced by mature DCs. This was also confirmed with HSV-1 pulsed DCs. In the primary B follicles of lymph nodes, FDCs are markedly reduced and poorly developed FDC that KiM4p negative and weakly positive for NGFR, CD21, and CD23 (60).

HIGM 3 is caused by hypomorphic mutations of CD40. Fontana et al. (57) observed normal differentiation of monocytes from 2 patients to iDCs in the presence of GM-CSF and IL-4 with normal expression of co-stimulatory molecules CD80 and CD86. Similar to HIGM1, these patients also demonstrate normal T cell proliferation by anti-CD3. TNF, LPS plus IFN, but not CD154 trimer-induced maturation of iDCs (expression of HLA-DR and CD83.

## 4 Dendritic cells in combined immunodeficiency with associated or syndromic features

The adhesion, synapse formation, and duration of interactions are critical for mature DC to induce activation of naïve T cells. *In vitro* experiments have demonstrated that mature DCs must remain stably attached to T cells (synapses) to induce T cell activation, whereas immature DCs make short and intermittent contacts with T cells (kynapses), and therefore, are unable to activate T cells (61). However, kynapses may induced T cell activation when antigen density is high, therefore allowing integration of signals over repeated serial contact interactions (62). ICAM-1-LFA interactions result in the formation of synapses resulting in T cell activation and differentiation of T cells to memory T cells, whereas absence of ICAM-1 may still form kynapses and activate T cells, however; failed to differentiate into memory T cells (63, 64).

Treg regulate synapse formation between DCs and CD4+ T cells by destabilizing stable contact between CD4+ T cells and DCs, thereby impairing the capacity of DCs to activate T cells. This regulatory effect, in part, is due to increased expression of CD39 and CD73, that hydrolyses ATP to ADP, AMP, and adenosine (65).

Actin cytoskeleton is essential for T cell signaling; it is required for TCR clustering in microclusters, and recruitment of signal complex to microculture. Several molecules are involved during synapse formation and its stability including integrin LFA-1, ICAM-1-3 on T cells, cytoskeletal regulatory proteins Wiskott-Aldrich syndrome protein (WASp), WASp interacting protein (WIP), and dedicator of cytokinesis 8 (DOCK8) (66–68).

DOCK proteins activate members of the Ras homolog gene family (Rho) of small guanine triphosphate binding proteins (GTPases), such as CDC42 and RAC, which integrate signals from the cell membrane to control pathways involved in actin polymerization and cytoskeletal rearrangement (69, 70). Failure of CDC42 activation due to missense mutation of DOCK8 is associated with clinical and immunologic features of DOCK8 deficiency. DOCK8 facilitates the accumulation of adhesion molecules and cytotoxic granules at immunologic synapses (71).

WASP protein regulates actin polymerization and cytoskeletal reorganization in hematopoietic cells and plays an important role in the functions of immune cells, including migration of DCs to lymphoid tissue, activation of T cells, and generation of regulatory T cells (62, 72, 73). CDC42 is also an important activator of WASp (74). Binding of CDC42 to WASp causes a confirmational change, which results in interacting with ARP2/3 complex and activate it, and initiates actin polymerization (75, 76).

### 4.1 Wiskott-Aldrich syndrome

Wiskott-Aldrich syndrome (WAS), a X-linked disorder caused by defective expression of WASP protein due to a mutation in *WASp* gene, is characterized by both cellular and antibody deficiency, increased susceptibility to infection, eczema, thrombocytopenia, and increased susceptibility to autoimmune diseases.

In *in vitro* experiments, DCs from patients with WAS display profound defects in migration, polarization, and translocation. These abnormalities have also been demonstrated *in vivo* in mice model (77). Pulecio et al. (78) in a *WASp*- mice using *in vivo* migration assay and time-lapse video microscopy, reported that the number of conventional DCs were similar to WT mice; however, antigen presentation by resident DCs to T cells to induce activation and development of memory cells was impaired. In adoptive transfer experiments, *WASp-* DCs were markedly impaired in their capacity to migrate to draining lymph nodes. Furthermore, correction of defective migration to lymph node did not rescue priming of CD8+ T cells. Therefore, suggesting that WASp expression in DCs is essential for T cell priming. Using imaging study, both *in vitro* and *in vivo* they demonstrated impaired stable interaction between WASp DCs and T cells. Catucci et al. (79), in a preclinical model of lentiviral-mediated gene therapy for WAS, demonstrated that *WASP* gene therapy resulted in migration of DCs in lymph nodes, and were able to efficiently induce antigen-specific activation of T cells.

### 4.2 DOCK8 deficiency

DOCK8 deficiency is a combined immunodeficiency characterized by eczema, recurrent respiratory as well as recurrent, persistent, and serious viral infections that are often resistant to treatment. In addition, these patients often experience recurrent sinopulmonary bacterial infections. DOC8 deficient patients and WAS patients have overlapping clinical manifestations, therefore, may suggest that they may share common pathogenic mechanisms.

DOCK8 plays a prominent role in both innate and adaptive immunity. Its role in innate immunity was reported in a murine model, which showed that dendritic cells rely on DOCK8 for trafficking from the skin to nearby lymph nodes and migrating dendritic cells deficient in DOCK8 cannot activate CDC42 resulting in severe impairments in polarization and migration (80). In addition, DOCK8-deficient patients have decreased circulating pDCs with impaired IFN-α production (81).

DOCK8 is also important in other aspects of innate immunity, such as the function and survival of RORγτ^+^ innate lymphoid cells (ILCs). DOCK8-deficient mice have decreased numbers of RORγτ^+^ ILCs in their GI tract. These ILCs display decreased IL-7 responsiveness and IL22 production, as well as a predisposition to apoptosis. The defect in ILCs reflects the importance of DOCK8 for optimal STAT3 activation and IL-22 production (82).

The immune abnormalities seen in patients with DOCK8 deficiency reflect the importance of DOCK8 in controlling both actin cytoskeleton-dependent and -independent immune responses. DOCK8 enables adaptive immune responses by several mechanisms. Through its regulation of the actin cytoskeleton, it facilitates the accumulation of adhesion molecules and cytotoxic granules at immunologic synapses (64, 72). Deficiency of these interactions may contribute to impaired B, T and NKT cell survival and long-lived memory responses in DOCK8-deficient patients. DOCK8 also regulates filamentous actin, LFA-1 and cytolytic granule accumulation in the cytotoxic synapse, processes critical for NK cell mediated cell death (71). When DOCK8 is suppressed in human NK cells, they demonstrate defects in natural cytotoxicity as well as specific activating receptor-mediated NK cytotoxicity (71). Without DOCK8 present to coordinate the actin cytoskeletal network, DOCK8-deficient T and NK cells undergo a form of cell death termed cytothripsis when migrating through confined shapes, such as collagen-dense tissues like the skin (71).

### 4.3 WASp interacting protein deficiency

WIP is a proline rich protein with homology to the yeast verpoline, a polarity developmental protein (83). WIP is expressed in a large number of tissues that is in contrast to WASp, which is expressed exclusively in hematopoietic cells. WIP associates with WASp and increases cellular polymerized actin and appearance of projections on the cell surface containing F-actin. WIP stabilizes WASp and also binds to actin (84). Several lines of evidence suggest a role of WIP in regulating actin cytoskeleton (85). T cells from WIP-deficient mice do not bind actin and show cytoskeletal and functional defects similar to T cells deficient in WASp. Janssen et al. (86) showed that WIP bridges DOCK8 to WASp and actin, which may provide a molecular mechanism for shared features of DOC8 deficiency and WAS.

The first primary immunodeficiency with WIP deficiency due to homozygous mutation of *WIPF1* gene was described in a female child presenting with clinical picture similar to WAS including recurrent infections, thrombocytopenia, eczema, and defective T cell proliferation and chemotaxis (87). However, platelet volume was normal. In this patient WAS sequence and mRNA were normal, but WASp expression was markedly decreased, therefore, suggesting an increased degradation of WASp, further supporting a role of WIP in stabilization of WASp. Since its first description, 7 more cases of WIP deficiency (often labeled as WAS2) have been reported.

### 4.4 DiGeorge syndrome

DiGeorge syndrome (DGS) is a congenital disorder due to hemizygous deletion of Chromosome 22q11.2. The syndrome is associated with varying degree of T cell deficiency ranging from normal, partially reduced, and complete absence of T cell numbers. Clinically, patients present with increased susceptibility to infections that subsides during early childhood (88); however, autoimmune diseases including thrombocytopenia, thyroiditis and idiopathic juvenile arthritis, tend to appear with increasing age due to an expansion of self‐reactive T cells and a reduced production of regulatory T cells (89, 90). Legitomo et al. (91) examined circulating mDCs and pDCs in 17 patients with DGS. They observed decreased proportions and number in DGS as compared to age-matched controls. Then they divided DGS patients according to vitamin D levels into those with normal, deficiency, and hypoparathyroid groups, most of the reduction in both mDCs and pDCs was observed in DGS with vitamin D deficiency and hypoparathroiroid groups. Since pDCs are major source of type I IFN with anti-viral activity, reduced pDCs may play a role in increased susceptibility to viral infections in DGS. DCs re important in regulating the development and induction of Treg, therefore, their reduction may be responsible for autoimmunity associated with DGS. However, number of subjects in their study are too small to draw a definitive conclusion.

### 4.5 Ataxia telangiectasia

Ataxia telangiectasia (A-T) is an autosomal recessive IEI caused by biallelic mutation of ATM gene encoding for ATM protein that belongs to the family of phosphoinositide-3-kinase like kinase (92). Patients with A-T clinically present with progressive neurodegeneration- primarily cerebellar atrophy, ocular and cutaneous telangiectasia, immunodeficiency, autoimmunity, radiosensitivity, and susceptibility to malignancies (93). ATM is a serine/threonine kinase that coordinate cellular responses to double stranded breaks. Many proteins are phosphorylated by ATM, resulting in cell-cycle control and activation of DNA repair systems. ATM is activated by oxidative stress, in the absence of double stranded break (94). ATM also appears to have immunoregulatory role. Westbrook and Schiestl (95) showed dextran-induced colitis was more severe in *Atm* ^−/−^ mice, and persistent activation of immune responses were observed in *Atm−/−* mice.

Wang et al. (96) investigated a role of ATM in the regulation of IL-23 in DCs. They demonstrated that Inhibition of ATM by highly selective antagonist KU55933 resulted in an increased IL-23 production in TLR-4-stimulated DCs and increased the level of Th17 responses from naive CD4^+^ T cells. In contrast, activating ATM signaling with ionizing radiation suppressed IL-23 responses. Shigematsu and colleagues (97) reported that exposure of DCs to low dose radiation (LDR) enhanced DC migratory capacity *via* increased expression of CCR7, and IL-12 secretion. The increased CCR7 expression and IL-2 secretion was due to the activation of ATM and downstream NF-kB. Yu et al. (98) also reported that LDR promotes ATM phosphorylation, and ATM specific inhibitor abolished LDR-induced upregulation of CCR7 and IL-12 production. Surprisingly, DCs have not been studied in patients with A-T.

### 4.6 Hyper IgE syndromes

#### STAT3 deficiency

The hyper IgE syndrome (HIES) is characterized by high IgE, eosinophilia, increased susceptibility to infections, especially chronic mucocutaneous candidiasis, respiratory tract infections with fungi and encapsulated bacteria, and skeletal abnormalities including decidual tooth retention, osteopenia, and scoliosis (99). Autosomal dominant form is caused by dominant negative signal transducer and activator of transcription 3 (STAT3) mutation. STAT 3 is a transcription factor that plays an important role in signal transduction for several cytokines including cytokines of γc receptor family. In mice, STAT3 has been shown to play a critical role in cell migration, proliferation, differentiation, and survival (100). Furthermore, STAT3 plays a regulatory role in DCs, and in the differentiation of naïve CD4+ T cells to TH17 cells (101). Saito et al. (102) reported that HIES patients carrying mutations in STAT3 or Tyk2 display normal *in vitro* differentiation and functions of TH1, TH2 and Treg cells in HIES, however, myeloid and monocyte-derived DCs were impaired in response to IL-10 to become tolerogenic (FoxP3+), and to upregulate PD-1 and ILT4 compared to control DCs. These impaired functions of DCs may be responsible for inflammation in HIES. Gutierrez-Hincapie et al. (103) reported circulating myeloid and plasmacytoid DCs in HIES comparable to controls. The expression of CD80 and CD86 on myeloid DCs in PBMNC-stimulated with LTA, and LPS in HIES was also comparable to controls. However, CD80 expression of CpG-stimulated PBMNC (plasmacytoid DCs) was lower in HIES as compared to controls, significance of this observation in unclear.

Korenfeld and colleagues (104) reported a reduced frequency of myeloid DCs, specifically cDC1 (CD141+) in patients with STAT3 gain-of- function mutations. Furthermore, they demonstrated impaired ex-vivo differentiation of CD14+ monocytes in to CD1a+ monocyte derived DCs. Furthermore, these DCs produced lower amounts of CCL22. CCL22 possess antimicrobial property and therefore, likely to contribute to increased susceptibility to infections in AD HIES. Furthermore, CCL22 regulates Treg interaction with DCs in the lymph nodes, therefore, a deficiency of CCL2 may explain the Treg dysregulation and autoimmune manifestations in patients with STAT3^gof^ mutation (105, 106).

### 4.7 HOIP deficiency

The linear ubiquitin chain assembly complex (LUBAC) plays pivotal roles in regulating lymphocyte activation, inflammation, and cell death. HOIL-1, SHARPIN and HOIP are the three components of LUBAC1. Patients with mutations in LUBAC catalytic subunits HOIP and in HOIL suffer from autoinflammation combined with immunodeficiency. This E3 ligase has been implicated in NF-κB signaling (107, 108). First case of HOIP was reported by Boisson et al. of a patient with multiorgan autoinflammation, combined immunodeficiency, subclinical amylopectinosis, and systemic lymphangiectasia, who had homozygous mutation in *HOIP*, the gene encoding the catalytic component of LUBAC (109). Oda et al. reported the second case of HOIP deficiency with novel compound heterozygous mutations in *RNF31* and distinct clinical and molecular features. Patient had specific antibody deficiency with normal immunoglobulins and suffered from severe bacterial, viral, and fungal infections, and autoinflammation was manifested by polyarticular juvenile idiopathic arthritis (110).

The pathogenesis of autoinflammation is not clear. Wu et al. (111) reported MyD88-dependent proinflammatory signal plays an important role in the pathogenesis of human autoinflammation associated with HOIP mutations. They demonstrated that although HOIP deficiency in DCs did not affect TNF-α-induced NF-κB activation, it enhanced TNF-α-induced apoptosis. Crossing HoipDC KO mice with TNFR1-knockout mice could not rescue the systematic inflammation, suggesting that the autoinflammation is not because of HOIP on TNF-α signaling. They observed that stimulation with LPS/TLR4 induced more apoptosis and significantly higher levels of IL-1α and IL-1β in HoipDC KO cells. Furthermore, MyD88 deficiency rescued the inflammatory phenotype in HoipDC KO mice.

## 5 Dendritic cells in predominantly antibody deficiency

### 5.1 X-linked agammaglobulinemia

In addition to B cells, DCs and monocytes also express BTK. Several investigators have studied DCs differentiation, maturation, and functions in XLA (112, 113) The expression of TLRs on moDC from XLA patients is comparable to controls (114). The differentiation and maturation of CD14+ monocytes into moDCs, as well activation by LPS/TLR4 as evident by upregulation of expression of MHC class II, CD83, CD80 and CD86 was normal (112, 113). Furthermore LPS-stimulated matured DCs (moDCs) from XLA patients produced similar amounts of IL-12 and IL-10 as controls (89). However, Lougaris et al. (113) reported impaired Cpg/TLR9-induced activation and production of IL-6, IL-12, TNF-a, and IL-10 by DCs from XLA. They also demonstrated that incubation of healthy DCs with Ibrutinib, the specific BTK inhibitor, resulted in an impairment of TLR9 responses similar to those observed in XLA patients, supporting a role of role of BTK in human DC activation. However, priming of naïve T cells by moDCs from XLA was comparable to controls. Therefore, BTK mutations do not influence the differentiation and maturation, and functions of moDCs in XLA.

Taneichi et al. (114) studied phenotypic maturation and cytokine production by TLRs stimulated moDC from XLA patients and healthy normal controls. The TLR expression in moDCs was analyzed by flow cytometry. TLR-mediated signaling in moDCs was evaluated for the phenotypic maturation based on CD83 expression and production of cytokines, such as TNF-α, IL-6 and IL-12p70. TLR levels in moDCs were similar between XLA and controls. TLR2, TLR4 and TLR7/8 ligands elicited less phenotypic maturation of moDCs from XLA patients than normal controls based on CD83 expression. Stimulation with TLR2, TLR4 and TLR7/8 ligands, as well as TLR3 ligand, resulted in significantly lower production of TNF-α, but neither IL-6 nor IL-12p70, by moDCs from XLA patients in comparison to normal controls. These findings suggest that BTK may thus be required for TLR signaling in moDCs. The impaired TLR signaling in moDCs may therefore be partly responsible for the occurrence of severe infections with bacteria and some viruses in XLA patients Sochorova et al. (115) analyzed whether BTK deficiency in XLA is associated with an impaired dendritic cell (DC) component or defective TLR signaling. They investigated the expression of TLRs 1 to 9 on myeloid DCs generated from XLA patients and evaluated their response to activation by specific TLR agonists. The numbers of circulating DCs in XLA patients were similar to healthy controls. BTK-deficient DCs have no defect in response to stimulation of TLRs 1/2, 2/6, 3, 4, and 5 but display a profound impairment of IL-6 and TNF-α production in response to stimulation by TLR-8 cognate agonist, ssRNA. These findings may provide an explanation for the susceptibility to enteroviral infections in XLA patients (115). pDCs from XLA patient upon stimulation with TLR-9 ligands (ODN A or C) induced IFN-α production similar to pDCs from healthy controls (116).

### 5.2 Common variable immunodeficiency

CVID is a heterogeneous disorder characterized by reduced IgG, IgA, and/or IgM, poor response to vaccines, and recurrent infections. Gene mutations account for only 15-20% of CVID cases, therefore, cause (s) of CVID in majority of cases remain unknown. A subset of CVID patients also display T cell dysfunction even though their numbers are normal in majority of patients. Among all IEI, DCs have been studied most extensively in CVID. Several investigators have studied DCs differentiation, maturation, and functions in CVID. Majority of the analyses have been on *in vitro* monocyte derived DCs. Bayry and colleagues (117) examined DCs from patients with CVID (diagnosed by impaired vaccine response and IgG subclass deficiency, and one patient with IgG4 and IgA deficiency). Therefore, these patients do not meet the criteria of diagnosis of CVID. They observed decreased expression of co-stimulatory molecules CD80 and CD83, and HLA-DR and CD40 on DCs from CVID patients. DCs from CVID patients were poor stimulators of T cells in allogeneic mixed lymphocyte cultures. Stimulation of CVID DCs with CD40L did not upregulate co-stimulatory molecules and IL-12 secretion was significantly lower than controls. Further, DCs from CVID patient produced low amounts of IL-10. Scott-Taylor and colleagues (118, 119) observed similar expression (number and intensity) of CD86 and HLA-DR between CVID DCs and healthy control DCs, but CD40 expression was reduced. However, following LPS stimulation upregulation of HLA-DR was significantly lower in CVID DCs as compared to controls; however, CD86 expression was not significantly different. CD83 expression with or without stimulation with LPS was similar in CVID and healthy controls. Intracellular IL-12p70, IL-6, and IL-1α before and following LPS stimulation were similar in both groups. They also observed that DCs from CVID stimulated T cells to produce cytokines similar to controls. However, DCs from CVID induced reduced T cell proliferation in allogeneic MLR.

Trujillo and colleagues (120) in PBMNC, observed no difference in circulating myeloid DCs in CVID as compared to controls. In contrast, circulating pDCs were markedly decreased in CVID as compared to healthy controls. However, Taraldsrud et al. (121) reported reduced numbers of both circulating myeloid DCs and pDCS in CVID. Trujillo et al. (120) observed that following stimulation of PBMNC with LPS, CpG, and LTA, expression of co-stimulatory molecules CD80 and CD86 was upregulated in both myeloid and pDC gated cells. No functional studies were performed on isolated DCs or monocyte derived DCs.

Cunnigham-Rundles and colleagues (116) reported that purified pDCs from CVID were phenotypically (CD4+CD11c-, MHC II+) similar to healthy controls. TLR9 levels in CVID patients (n=4) were comparable to healthy controls (n=4). No polymorphisms in TLR9 were observed. However, when purified pDCs from CVID patients were stimulated with ODN A or C (TLR-9 ligands) significantly less IFN-α was produced as compared to healthy controls. Yu et al. (99) also observed significantly less IFN-α production from purified pDCs from CVID patients upon stimulation with loxoribine (TLR7 ligand). These data would suggest a defect in TLR7 and TLR9 signaling in CVID. In contrast, Taraldsrud et al. (121) observed similar levels of intracellular IFN-α and IL-12 in DCs following stimulation with TLR9 ligand. Since later investigators did not purify pDCs, other cells in PBMNC may have compensated for defects observed by Cunningham-Rundles et al. (116).

## 6 Dendritic cells in disorders of immune regulation

### 6.1 APECED

Autoimmune polyendocrinopathy, candidiasis, ectodermal dystrophy (APECED) syndrome is a monogenic IEI caused by mutation of the autoimmune regulator (*AIRE*) gene. APECED is characterized by an unusual susceptibility to mucocutaneous candidiasis and multiorgan autoimmune diseases (122). APCED established a role of AIRE in immune tolerance. Since AIRE is expressed predominantly in thymus medulla, it plays an important role in central tolerance. In the periphery, AIRE is expressed in dendritic cells (DCs) and therefore, appears to be play an important role in peripheral tolerance (123). T follicular helper (T_FH_) cells are critical in germinal center formation, and B cell differentiation as well selection and activation of autoantibody producing B cells (124). T_FH_ cells are increased in human and experimental models of autoimmune diseases (125). A role of AIRE in negative regulation of T_FH_ cells has been demonstrated by increased T_FH_ in AIRE-deficient mice (126). Follicular DCs with high ICOS expression and IL-27 production play an important role in inducing T_FH_ cells. Zou et al. (126) have shown that Aire overexpressing DCs (DC2.4) inhibit T_FH_ cell differentiation *via* suppression of ICOSL and IL-27 expression. Li et al. (127) reported delay in the development of type I diabetes in mice by transplantation of AIRE overexpressing bone marrow derived DCs.

### 6.2 CTLA-4 deficiency

CTLA-4 Haploinsufficiency with Autoimmune Infiltration’ (CHAI) **i**s characterized by lymphadenopathy and/or splenomegaly, autoimmune diseases including autoimmune cytopenias, type 1 diabetes, autoimmune thyroiditis, hepatitis, arthritis, and autoimmune skin disorders lymphocytic infiltration of multiple non-lymphoid organs (predominantly gut, lungs, and brain), recurrent respiratory tract infections, and a progressive loss of circulating B cells and/or immunoglobulin levels, and accumulate CD21^lo^ B cells over time (128, 129). CTLA-4 is highly expressed on Treg cells. Tregs mediate their suppressive effect by several different mechanisms. One of the mechanisms is CTLA-4-dependent trogocytosis of CD80 and CD86 molecules. Therefore, one would expect upregulation in co-stimulatory molecules on DCs in CTLA-4 deficiency. Therefore, reduced Treg function may explain autoimmune diseases in CTLA-4 deficiency. Surprisingly, there is not a single study of human CTLA-4 deficiency that has investigated DCs.

### 6.3 Autoimmune lymphoproliferative syndrome

Autoimmune lymphoproliferative syndrome (ALPS) is a disorder of immune regulation characterized by non-malignant proliferation of lymphocytes with lymphadenopathy/hepatomegaly, increased double negative T (α-β DNT cells), impaired apoptosis of T cells, and increased circulating levels of FAS ligand (CD95L), IL-10, IL-18 (130, 131). ALPS Ia is associated with CD95 (FAS) mutation (132), whereas ALPS Ib is linked to inherited CD95L mutation (133). ALPS II is associated with inherited caspase 10 mutation (134), and inherited caspase 8 mutation is associated with ALPS (135). DCs have been studied in ALPS II associated with inherited caspase 10 mutation. Caspase-10 is known to induce apoptosis in MCF-7 cells (136); however, its significance was unclear until recently, when Wang and colleagues (134) described a family with ALPS II associated with independent missense mutation of caspase 10. They studied DCs apoptosis *via* different death receptor pathways. They demonstrated that activated T cell-induced apoptosis of DCs is mediated *via* TRAIL, and CD95 and TNF-α had minimal effect on DC apoptosis. DCs from patients with ALPS II displayed impaired TRAIL-induced apoptosis as compared to DCs from ALPS I patients and healthy controls. Furthermore, there was an accumulation of CD3+ T cells and DCs in T cell areas of the lymph nodes, that was not observed in ALPS I patients. Therefore, combined defect of T cells and DCs may be responsible for ALPS II phenotype.

### 6.4 Hemophagocytic lymphohistiocytosis

Hemophagocytic lymphohistiocytosis (HLH) is an inflammatory disorder characterized by episodes of extreme immune activation associated with hypercytokinemia, accumulation of IFN-γ producing T cells and activation of macrophages (137, 138). In humans, familial hemophagocytic lymphohistiocytosis (FHLH) is caused by mutations in *perforin* or other genes including *munc 13-4* that affect perforin-mediated cytotoxicity (139–141). Chen et al, demonstrated that antigen-specific activated CD8+ T cells induce apoptosis of DCs in mice by both perforin and Fas-dependent manner (142). Chen et al, in transgenic mice expressing the baculoviral caspase inhibitor, p35, in DCs (DC-p35) observed that DC-p35 mice displayed defective DC apoptosis, resulting accumulation of DCs and chronic lymphocyte activation and systemic inflammatory and autoimmune manifestations (142).

Perforin knockout mice (perforin -/-) with LCMV infections also display impaired T cell cytolytic activity and uncontrolled activation of IFN-γ producing CD8 T cells, and inflammatory manifestations resembling FHLH (143, 144).

Chen et al. (142) demonstrated that perforin -/- and Fas -/- CD8+ T cells were weaker in killing antigen pulsed DCs as compared to CD8 T cells from wide type CD8 T cells, suggesting that perforin and Fas may synergize. In adoptive transfer experiments, co-transfer of wild type antigen-activated T cells with DC resulted in reduced number of DCs in draining lymph nodes, consistent with killing of DCs by antigen-activated T cells. Furthermore, in perforin -/-DC-Fas-/- mice significant increase in spleen and lymph nodes was observed as compared to wild type mice. These mice also had increased conventional and plasmacytoid DCs, and increased expression of CD80 and CD86 demonstrating that more active DCs were present in perforin-/- as compared to wild type mice. In contrast, no change in macrophages was observed. In perforin-/-DC-Fas-/- mice, increased IFNγ production by CD8+ T cells was observed but not in wild-type, DC-Fas-/- or perforin-/- mice. Interestingly, CD4+ T cells, DCs, macrophages, or NK cells did not show increased intracellular IFNγ. In perforin-/-DC-Fas-/- mice, lung, liver and kidney display evidence of inflammation as evident by severe perivascular lymphocytic infiltration. These mice also developed anemia and thrombocytopenia. These investigators also demonstrated that CD8 + T cells are the main effectors of inflammation in these animals. Therefore, antigen activated CD8+ T cells are potentially the predominant cell type that restrict the life span of antigen presenting DCs. In deficiency of perforin in T cells and Fas in DCs result in increased survival of DCs and abnormal and prolonged activation of T cells resulting in inflammation.

## 7 Congenital defects of phagocyte number and function

### 7.1 GATA-2 deficiency

GATA family transcription factors play an important role in the differentiation of hematopoietic stem cells (HSC) into precursors of special lineages (145). These include GATA-1, GATA-2, and GATA-3. GATA-2 is highly expressed in HSCs, multipotent hematopoietic progenitors, erythroid precursors, megakaryocytes, eosinophils, and mast cells, and functions early in hematopoiesis where it is required for survival and expansion of HSCs and multipotent progenitor cells (146).

In 2011, three group of investigators independently (147–149) described heterozygous *GATA-2* germline mutations causing three overlapping clinical entities [a] familial MDS/AML, [b] Emberger syndrome, and [c] an immunodeficiency due to monocytopenia characterized by *Mycobacterium avium* complex (MonoMAC)/dendritic cell (DC), monocyte, B- and natural killer (NK)-lymphoid deficiency (DCML).

In patients with GATA-2 deficiency syndrome, monocyte, B cell, NK cell, and DCs are markedly diminished or absent, whereas neutrophil, macrophage, and T cell populations remain unaltered (147, 148). Reduced proportion of HLA-DR+CD14- CD16- CD11c+ cells (cDC1) and often pDC deficiency, and Progressive hypogammaglobulinemia are observed. Because of markedly reduced numbers of monocytes and DCs, functional studies have not been performed. Clinical features include recurrent infection (especially atypical mycobacterial infections, EBV, Pneumocystis jirovecii and recurrent HPV-related warts) and hematological malignancies (MDS/AML), pulmonary alveolar proteinosis (PAP), lymphedema, and sensorineural deafness. Rheumatological manifestations have also been reported (150).

### 7.2 Chronic granulomatous disease

Chronic granulomatous disease (CGD) is a rare primary immunodeficiency disease characterized by recurrent bacterial, fungal infections, and granuloma formation in various organs and tissues. CGD is caused by defects in genes encoding the subunits of the nicotinamide adenine dinucleotide phosphate (NADPH) oxidase complex, resulting defective intracellular killing of microbes without any effect on their phagocytosis, often (151–153) associated with inflammatory and autoimmune manifestations (154–156).

NADPH oxidase has shown to play an important role in regulating in antigen processing and cross presentation by DCs to antigen specific CD8+ T cells. Amogerina group (151–153), in both *in vivo* and *in vitro* using gp91phox deficient mice, demonstrated that DCs from gp91phox- mice were impaired in antigen degradation and MHC class I presentation to CD8+ T cells and activation of CD8+ T cells (148–150, 156).

Rybeca et al. (157) in gp91phox- mice demonstrated impaired proteolysis of antigens in phagosome. Amogerina group also reported reduced *in vitro* MHC-I restricted antigen presentation to CD8+ T cells by DCs from CGD patients and DCs from healthy donors treated with oxidase inhibitor; inhibitor did not alter phagocytosis by DCs (158).

### 7.3 Leucocyte adhesion deficiency

There are three different forms of Leucocyte adhesion immune deficiencies (LAD). LAD I (LAD1) is the most common rare autosomal recessive disorder characterized by delayed separation of the umbilical cord, leukocytosis, recurrent severe bacterial and fungal infection and not viral infections, delayed wound healing with defective neutrophil mobility and without pus formation (159). LAD-1 is caused by germline mutation of *ITGB2* gene encoding for CD18, the subunit of β2 integrin (160, 161). B2 integrins are heterodimeric receptors consist of α (CD11a-CD11d) and β (CD18) subunits. Lymphocyte function associated antigen-1 (LFA-1) that is comprised of CD11a/CD18. It is the only β2 integrin that is expressed on all types of leucocytes including DCs (162). LFA-1 binds to intracellular adhesion molecules (ICAMs) and this interaction is critical for immunological synapse formation between APC such as DC and T cells (163). LFA-1 plays an important role lymphocyte migration from blood to inflammatory site (164). Patient with LAD-1 are also at risk for developing autoimmune diseases (165), which suggest impaired peripheral tolerance that is predominantly mediated by DCs (166). Bednarczyk et al. (167) studied the role of β2 integrins on DCs cytokine signaling and inflammation and induction of autoimmune disease in a murine model. They generated a mouse strain with a floxed CD18 gene locus to knockdown β2 integrin in DCs. Splenic DCs from these mice showed increased cytokine production in response to stimulation with various TLRs. Furthermore, they showed increased activation of STAT 1, 3, 5 and impaired expression of suppressor of cytokine signaling. In MOG-induced experimental autoimmune encephalitis model, mice with a DC-specific β2 integrin knockdown displayed a delayed onset and attenuated course of the disease. No similar studies of DCs in LAD-1 deficient patients have been reported.

## 8 Defects in Innate and Intrinsic Immunity

### 8.1 Mendelian susceptibility to mycobacterial disease

Mendelian susceptibility to mycobacterial disease (MSMD) is a rare inherited condition characterized by selective predisposition to clinical disease caused by weakly virulent mycobacteria. However, rarely infections with more virulent *Mycobacterium tuberculosis, Salmonella typh*i, infections by various intracellular bacteria, fungi, parasites, and viruses have been reported. Mutations in 9 different genes involved in IFNγRI-IL-12/23 signaling. These include *IFNγRI, IFNγRII, STAT1, IL12β, IL-12RβI, IRF8, ISG15, CYBB*, and *NEMO* (reviewed in 150). IL-12Rβ1 deficiency is the most common genetic etiology of MSMD, closely followed by IFN-γR1 deficiency.

Mutations with both recessive and dominant inheritance patterns result in IFN-γR1 deficiency, most recessive IFN-γR1 deficiencies result from complete loss of IFN-γR1 expression on the cell surface therefore, complete loss of IFN-γ responsiveness.

Dotta et al. reported transient decrease in circulating mDCs and pDCs in patients with IFNγR1 deficiency (168). The acute phase of the mycobacterial infection associated with a marked reduction of both circulating mDCs and pDCs, and decreased pDCs in the lymphoid tissue. DCs subsets progressively increased and normalized after the end of the 18-months therapy. In addition, *in vitro* maturations of DCs appeared to be normal. Altered DCs traffic or increased apoptosis of DCs because of defective IFNγR1-mediated signaling has been proposed as possible mechanisms for depletion of DCs during mycobacterial infection.

### 8.2 IRF-8 deficiency

Interferon regulatory factor 8 (IRF8) is a member of the IRF family of transcription factors that binds to IFN-stimulated response elements (ISRE) and regulate the expression of genes stimulated by IFN (169). IRF-8 is required for maintaining cDC1 identity and maintaining cDC1-associated chromatin accessibility. Furthermore, IRF8 prevents conversion of cDC1 into cDC2-like cells (170). IRF8 deficiency induces the transcriptional, functional, and epigenetic reprogramming of cDC1 into the cDC2 lineage (170).

IRF8 is expressed in macrophages and dendritic cells and mutations of the human IRF8 gene results in two different immunodeficiencies. Autosomal recessive (AR) complete IRF8 deficiency that is caused by bi-allelic K108E mutation (171, 172). This immunodeficiency is characterized by a complete absence of CD14+ and CD16+ circulating monocytes, CD11c+ DCs and CD11c+/CD123+pDCs, and severely impaired IL-12 and IFN-γ production. Patients with biallelic mutations of IRF-8 clinically present with increased susceptibility to infections including disseminated BCG disease, oral candidiasis, and viral infections.

Bigley et al. (173) reported a child with compound heterozygosity of 2 defective IRF8 alleles, R83C and R291Q. Patient presented with early history of recurrent severe viral respiratory tract infection with influenza H1N1, rhinovirus, and mycoplasma. Profound depletion of CD141+ classical monocytes, CD161+ nonclassical monocytes, CD123+ pDCs, CD141+ cDC1s, and CD1c1 cDC2s were observed. However, tissue macrophages were normal. Absent IL-12 production markedly reduced IFN-γ, IL-6, IL-10, and TNF-α production by PBMNC; however, no functional studies were performed on isolated DCs

Autosomal dominant partial form of IRF8 deficiency is due to mono-allelic mutation (T80A) of IRF8 (172, 173). The T80A mutation has pleiotropic effects on IRF8 function. These patients have normal numbers of circulating lymphocytes, granulocytes, and monocytes, including two subsets of monocytes (CD14+ CD16−, and CD16+ and CD14dim). However, the main subset of human mDCs (DR+ CD11c+ CD1c+, or mDC1) are lacking.

### 8.3 CARD 9 and MyD88 deficiency

Caspase-associated recruitment domain protein 9 (CARD-9) plays a critical role in regulating innate immune response, especially acts as a regulator of Dectins and other molecules involved in recognition of fungal pathogens (174). CARD-9 deficiency, a rare inborn error of immunity, is characterized by superficial and deep fungal infections, and associated with defective cytokine production in response to fungal ligand (175–177). Myeloid differentiation primary response protein 88 (MyD88) is a cytosolic adaptor protein that is essential for most Toll-like receptors (TLRs), except TLR3 signaling to activate canonical pathway of NF-κB resulting in transcription of several proinflammatory cytokines. Patients with autosomal recessive MyD88 deficiency otherwise healthy, present with life-threatening, often recurrent pyogenic bacterial infections, including invasive pneumococcal disease. These patients display normal resistance to other microbes (178).

In a patient with homozygous mutations in MYD88 and CARD-9, who presented with pyogenic bacterial infection, and persistent EBV viremia, Chiriaco et al. (179) observed impaired differentiation of iDCs into mDCs. However, the proportions of myeloid DCs and plasmacytoid DCs were normal.

## 9 Autoinflammatory disorders

Autoinflammatory disorders (AIDs) are more recently incorporated in primary immunodeficiency/inborn errors of immunity. They are characterized by unprovoked inflammatory episodes in the absence of autoantibodies. Surprisingly, there are very few studies of DCs in AIDs.

### 9.1 Familial Mediterranean Fever

Familial Mediterranean Fever is the most common monogenic AID that is characterized clinically by episodic recurrent febrile episodes with serositis, synovitis, and erysipelas-like erythema, which usually subside within 24-72 h. The disease is caused by the *MEFV* mutations encoding for “Pyrin”. Funk and colleagues (180) reported extensive studies in patients with FMF. They studied 25 patients of whom 14 had heterozygous M694V mutation. However, data were not analyzed according to mutation, and not all analyses were performed on all cases. No significant difference was observed in circulating DC1, DC2 and pDCs in FMF patients (n=14) as compared to matched healthy controls. *In vitro* CD1a+HLA-DR+CD14^low^ immature MoDCs (ImMoDCs) from FMF patients were generated in numbers comparable to healthy controls. ImMoDCs from FMF patients display characteristics of mature MoDCs as evident by increased expression of CD83, CD86, HLA-DR, increased response to CCL19 signaling, response to TLR4 signaling, and increased capacity to stimulate T cell proliferation. In contrast, mannose receptors CD206 and CD209 (DC-SIGN) are significantly downregulated. It would be of interest to investigate inflammatory role of DCs in AIDs.

In summary, we have reviewed DCs in a number of categories of IEI. A detailed discussion is presented in disorders where significant data are available e.g. antibody deficiency diseases, combined immune deficiency diseases, disorders of immune regulation, and disorders of immune synapse formation including WAS, DOCK8 deficiency and WIP deficiency. Given the pivotal role of DCs in immune responses, it is essential that DC biology (phenotypic and functional) be studied in-depth in larger number of IEI to decipher their role in immunological abnormalities and clinical manifestations of IEI. It is also unclear whether different mutations in a particular IEI have different effect on the functions of DCs.

## Author contributions

AA wrote the Introduction and edited the manuscript. SG wrote DCs in IEI and edited the manuscript. All authors contributed to the article and approved the submitted version
